# Beware of physiology: Anthropomorphism as a simplification mechanism for mastering complex human-machine interfaces

**DOI:** 10.1371/journal.pone.0321580

**Published:** 2025-04-15

**Authors:** Axel Roques, Yannick James, Nicolas Vayatis, Pierre-Paul Vidal

**Affiliations:** 1 Université Paris Cité, Université Paris Saclay, ENS Paris Saclay, CNRS, SSA, INSERM, Centre Borelli, F-75006, Paris, France; 2 Laboratoire GBCM, EA 7528, Conservatoire National des Arts et Métiers, Paris, France; 3 Thales AVS, Osny, France; 4 Plateforme d’Etude Sensorimotricité, CNRS UAR2009, INSERM, Université Paris Cité, Paris, France; 5 Institute of Information and Control, Hangzhou Dianzi University, Hangzhou, China; The University of British Columbia, Australia

## Abstract

Humans’ remarkable technical reasoning skills have led to the development of increasingly sophisticated tools. In particular, society has welcomed the advent and democratization of machines that produce effects through indirect causal chains. Intuitively, perfect mastery of such systems should require a detailed understanding of their underlying processes. This raises the question of the boundaries of human cognitive abilities in the context of tool use. In other words, can the human brain integrate the characteristics of any tool, or are there inherent limitations? The present study investigates the potential limits of human tool-use when faced with a complex human-machine interface. To this end, professional helicopter pilots conducted realistic flights in a high-fidelity helicopter simulator. A comprehensive analysis was then conducted on the flight trajectories, the directions of movement of their primary flight command, and the tilt of their head as a function of the aircraft’s tilt in the roll plane. Our findings suggest that helicopter pilots severely restrict the capabilities of their aircraft. This simplification mechanism confines the operational range of the helicopter to conditions that elicit sensory inputs comparable to those experienced in everyday life. Our results further indicate some level of prediction regarding the sensory consequences of the motor-to-mechanical transformations. In sum, we postulate that control over complex human-machine interfaces is achieved by simplification through *anthropomorphism* in order to facilitate sensorimotor integration. These considerations have implications for the design of human-machine interfaces and raise safety concerns when interacting with highly sophisticated systems.

## Introduction

The critical importance of tools in human evolutionary history is evidenced by the astonishing ability of our brains to adapt when using them. Early studies by Maravita and colleagues [1,2] have highlighted how prolonged tool use alters visual-tactile spatial integration. Their research showed that experience with using simple tools led to functional changes in the body schema, a plastic neural representation of the body [3], effectively transforming the external object into an extension of our limbs. In some cases, the end-effector is not only *distalized* from the limb to some part of the tool [4] but truly embodied as they are actively and directly involved in somatosensory processing [5]. Through a dynamic update of the corporeal representation, which depends on the tool’s morphology and its particular sensorimotor constraints [6], tools shape the peripersonal space, i.e., a region of space proximal to the body that is the foundation for multimodal sensory integration and motor activity [7]. Such changes may result in temporary modifications in behavior, as one’s ability to act on their immediate environment is influenced by the characteristics of the tool [8]. For instance, the evaluation of distance and size are distorted when using a reach-extending tool [9].

However, effective control of tools requires a profound understanding of *motor-to-mechanical* transformations [10], i.e., the relationship that binds the movement of the body to the actions of the tool [4]. Accordingly, Martel et al. [11] suggested that tool mastery depends on the capability of incorporating the tool into the body schema; that is the capacity to embody the tool motorically [12]. To promote the motor embodiment of external objects, the sense of agency is paramount [13]. This sense corresponds to the experience of controlling external events and their sensory outcomes through one’s actions [14]. Traditionally, agency was thought to emerge when the predictions of an individual’s internal models aligned with the sensory information experienced when interacting with a tool in its environment. While this comparator model of agency was found insufficient – multiple factors contribute to the sense of agency, such as the recency of the last use [15] or the degree of expertise [8] – the aforementioned comparison mechanism is still believed to be one of its key mediators [16]. The sense of agency cannot be present if the mapping between the body and the tool is undecipherable, and thus, tool proficiency cannot develop.

Fortunately, and in sharp contrast to many other species that display tool-use behavior, humans excel in the use of complicated machinery that transforms motor commands into different mechanical outcomes [17]. Our unique technical reasoning skills have led to an increase in the efficiency and complexity of tools and techniques, a phenomenon known as cumulative technological culture (CTC) [18,19]. In the context of the CTC, expertise may be partly attributed to our extraordinary aptitude for establishing a sense of agency, even with highly complex systems that produce effects through indirect causal chains, or even when the body experiences conflicting sensory inputs [20]. Evidence suggests that some form of embodiment – in the broad sense, i.e., “the extra-personal experience of operating a tool as an extension of the body” [21] – can be achieved: experienced drivers were found to underestimate the distance to targets when sitting in an immobile car compared to when sitting on a chair [22]. These new insights into human cognition challenge the classical approaches, emphasizing the dynamic interplay between humans, tools, and the environment in our cognitive processes. They underscore how the latter may extend beyond bodily limitations, integrating tools, technologies, and interactions with the world around us. Nevertheless, little is known about the potential limits of this integration. To the best of our knowledge, no prior works have tried to investigate whether and how the human brain manages to effectively incorporate complex tools.

In this article, we propose to reflect on the concepts of tool-use in advanced human-machine interfaces. More precisely, we investigate whether there exists boundaries in human cognitive abilities when controlling a complex system. That is, can the human brain integrate the properties of any tool? To address this question, realistic flight scenarios were conducted by professional helicopter pilots in a high-fidelity helicopter simulator. As would be expected, all pilots were able to efficiently maneuver their aircraft and complete their mission. Nonetheless, our results show a substantial restriction in the operational range of the helicopter within an anatomically sound range. This suggests that pilot may adopt a simplification strategy that promotes sensorimotor integration. The present and future implications of human tool-use given the CTC, i.e., considering the exciting prospects offered by our continuing ability to innovate and adapt tools to our ever-changing needs, are discussed. By exploring these issues, we hope to contribute to a better understanding of the complex relationship between human operators and advanced technological interfaces.

## Material and methods

The Institutional Review Board Paris Descartes (CERES N°2017-35 dated 23/5/2017) approved the experimental protocols following the 1964 Helsinki Declaration. Data were collected from June 2017 to January 2018. Before testing, all participants gave their informed written consent. The authors did not have access to information that could identify individual participants during or after data collection.

### Experiments

The experiments involved in this study were described in previous works [23]. We refer the interested reader to this reference for additional details on the experimental protocols.

#### Helicopter flight scenarios.

Pilots conducted two flights in a highly realistic helicopter simulator (see S1 Fig for more information on the experimental apparatus). Both missions consisted of typical pilot duties, the first being a recon mission and the second a medical evacuation.

Motion of the simulated helicopter was sampled at 25 Hz. The position and the force exerted on the flight commands were also measured at 25 Hz. The pilot’s head movements were measured at 100 Hz with an inertial-optical hybrid head tracker (IS-1200 + HObIT System, InterSense).

#### Control experiment.

Participants were asked to visually explore their environment while sitting on a chair in an office. No specific instructions were given regarding the expected movements. Each subject conducted 3 repetitions of 3 minutes of visual exploration.

Head movements were measured at 120 Hz with an IMU (Movella DOT, Movella).

#### Participants.

Nine professional helicopter pilots (39.0 ± 5.7 years old, all male) with different backgrounds (police, army and air force) conducted flights in the simulator. Pilots had different expertise levels (median (Q1-Q3): $1520\left(991-2188\right)$ hours of real flight experience, median (Q1-Q3): $29\left(26-54\right)$ hours of simulator experience). All pilots had flown at least fifty hours in the twelve months preceding the experiment. Due to measurement issues, only data from at most 8 pilots (38.5 ± 5.9 years old) were usable. A visual illustration of pilot information can be found in S2 Fig. Eleven subjects (33.8±13.5 years old, 1 female) participated in the control experiment. Because of limited pilot availability, these participants were distinct from the pilots.

All participants were healthy and had no known medical history that could impair the experiments.

### Data analysis

All data analysis was performed in the Python programming language. The *SciPy* library [24] was used for all frequency and statistical analyses.

#### Aircraft motion.

The trajectory of the simulated helicopter was segmented into a series of distinct flight phases. Within each phase, the aircraft maintained a consistent direction of travel. Trajectory data was first subsampled to 1 Hz and standardized to facilitate analysis. The 2-dimensional horizontal motion of the helicopter was then used as the input of a change point detection algorithm [25]. Since the number of expected change points is unknown, a linearly penalized segmentation (Pelt) method was used with a minimum distance between change points of two seconds, in order to avoid generating short, artefactual segments.

To find the principal direction of motion in each segment, the 3-dimensional trajectory of the aircraft was recovered. Principal component analysis (PCA) was then applied to each segment. The first component, which constitutes a representative direction of the trajectory, was computed in the terrestrial reference frame and then projected into the reference frame of the aircraft. To do so, the first 10% of the segment were used to define the initial direction of movement and the first PCA component was simply compared to this reference direction to obtain 3-dimensional motion offsets. S3 Fig illustrates this process.

#### Manipulandum motion.

The movement of the cyclic stick, i.e., the joystick that controls the pitch and roll orientation of the helicopter, was segmented. The consecutive positions of the manipulandum were clustered based on an angular deviation threshold. A collection of 2-dimensional vectors was first generated by differentiating the position of successive samples. The angle between each vector and its predecessor was then computed. If the angle was less than a fixed threshold $\tau_{angle}=10$ degrees, a new vector was computed as the sum of the two. This new vector represents the principal direction of motion of the segment. When the angle between the representative vector of a segment and a new vector exceeded the threshold, the current segment was terminated and a new segment was started. An example of this method can be found in S4 Fig.

Once segments were identified, the direction of motion was projected onto the 2-dimensional roll-pitch plane and oriented with respect to the positive roll-axis. Segments shorter than 200ms as well as null segments were removed.

#### Vestibular input statistics.

The properties of the vestibular inputs experienced by the participants in the different experiments were inferred from the measurement of their head movements as in [23]. Briefly, the angular velocity of the head was projected onto the planes of the semicircular canals – the left anterior, right posterior (LARP), right anterior, left posterior (RALP), and yaw (YAW) planes – using the adequate rotation matrix. Probability density functions were derived from the velocity distributions with a Gaussian-kernel density estimate. Power spectral densities were computed with Welch’s average periodogram method with parameters nfft=2048 and a Bartlett window of $n_{window}=2048$ samples. The total power in each spectrum was computed as the area under the spectral density curve with the trapezoidal integration rule.

#### Statistical analysis.

All statistical analyses were performed with either the Mann-Whitney U test or the Wilcoxon signed-rank test to measure, respectively, whether the distributions underlying two samples were the same or whether two related paired samples came from the same distribution.

## Results

### Machine trajectory is highly restricted

**Fig 1:**
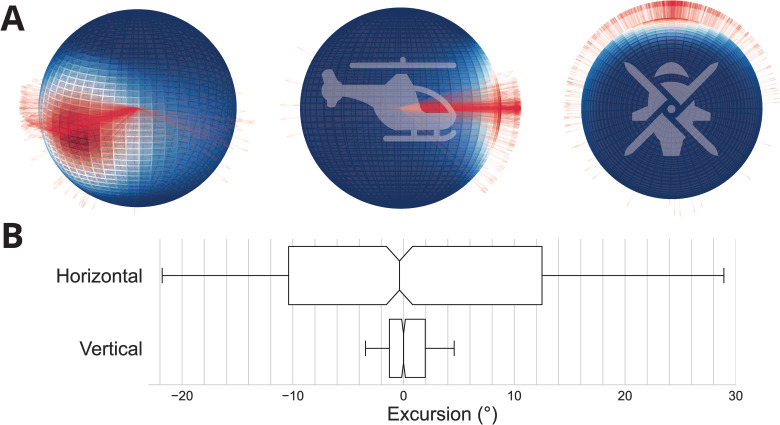
Helicopter trajectory characteristics. (**A**) Principal directions of movement of the helicopter trajectories, for all pilots and all scenarios. Trajectory directions are computed with respect to the helicopter’s initial orientation, as depicted in the middle and right spheres. Trajectories are represented as vectors. The number of trajectories along any particular direction is color-coded in the intensity of the vector and also represented in the spherical density distribution. (**B**) Box plots of the excursions of the helicopter in the horizontal and vertical planes, for all pilots and all scenarios. The notches represent the confidence interval around the median. Whiskers represent 70% of the data.

Aircraft movement was examined. To that end, the trajectories obtained in the two scenarios were segmented (see Material and methods). In each segment, the most representative direction of motion was computed and represented in the reference frame of the helicopter (Fig 1).

We found that compared to the theoretical range of motion, the range observed in practice is remarkably small. Indeed, the flight mechanics of a helicopter have been designed to allow a high degree of flexibility in piloting. This flexibility encompasses a wide range of trajectories whose directions collectively cover a nearly perfect sphere. Although we have observed that some pilots occasionally dare to exploit these capabilities, such as a backward takeoff, for example, Fig 1, A indicates that these practices constitute a minority. Most of the trajectories are confined to a cone whose axis is aligned with the horizon and orthogonal to the vertical (see the density distribution in Fig 1, A and the median values of the box plots with their respective confidence intervals in the horizontal and vertical planes in Fig 1, B).

The cone that characterizes the majority of motion directions is approximately right elliptical, i.e., its axis passes perpendicularly through the center of its elliptical base. These geometric properties reflect an asymmetry between the horizontal and vertical planes of motion, and a relative symmetry within each plane (Fig 1, B). More specifically, our results suggest greater piloting freedom in the horizontal plane compared to the vertical plane across both scenarios (p<0.01, comparison of the inter-quartile range, Wilcoxon test).

Statistical analysis reveals no significant difference in the total excursion range in the horizontal plane between the two flights but a significant difference in the vertical plane (p>0.05 and p<0.1 respectively, comparison of the inter-quartile range, Mann-Whitney U test). This trend is reversed when considering the distribution of excursions for all pilots: no statistical difference exists in the distribution of trajectory excursion in the vertical plane between the two flights but a significant difference exists in the horizontal plane (p<0.01 and p>0.1 respectively, Mann-Whitney U test). These fluctuations in the shape and extent of the distributions in both planes of motion can be explained by scenario-dependent factors, such as the goal of the mission, the weather, etc. On average, however, pilots did not seem to favor any particular direction of motion, as evidenced by the relative symmetry around zero in the two planes (Fig 1, B).

### Manipulandum motion is strictly constrained

**Fig 2:**
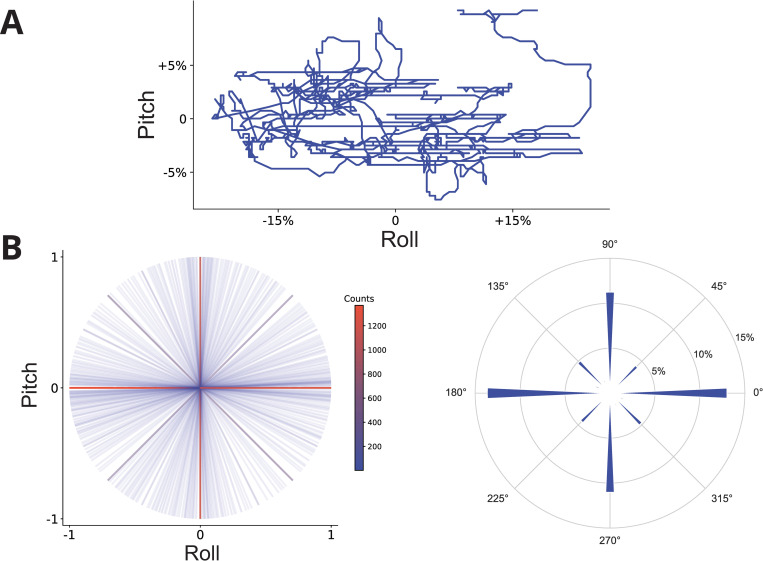
Movement characteristics of the manipulandum (cyclic stick). (**A**) 1.5-minute excerpt of the motion of the manipulandum for a single pilot in a single scenario (third pilot, first scenario). The manipulandum’s position is represented as a proportion of the total possible excursion in the pitch and roll planes. (**B**), left: Normalized principal directions of movement of the manipulandum in 10 000 randomly selected segments, amongst all pilots and scenarios. The color represents the number of movements along any particular direction. (**B**), right: Angle between the principal direction of movement in all segments and the positive horizontal axis. Data are binned in 5° bins and correspond to all pilots and all scenarios. The radial axis represents the proportion of movements angled at any particular value with respect to their preceding segment.

The trajectory of the helicopter is a direct consequence of the pilot’s actions on the flight controls. In light of the aforementioned findings on the motion of the aircraft, an analysis was conducted on the movements of the cyclic stick, the joystick that controls the primary rotor system (Fig 2).

We found that the projection of the manipulandum’s motion onto the 2-dimensional pitch and roll plane showed surprisingly linear paths (Fig 2, A). When continuous movements of the cyclic were segmented into canonical segments (see Material and methods), the principal directions of movement across all segments were largely restricted to the cardinal directions, along with the main diagonals (Fig 2, B, left). Even though diagonal movements are theoretically possible and were effectively carried out in practice (faint blue lines in Fig 2, B, left), they remain largely outnumbered by the others. This effect is formally quantified on the right panel of Fig 2, B, which depicts the proportion of movements in all directions for all pilots and scenarios.

### Head vs. machine movements

**Fig 3:**
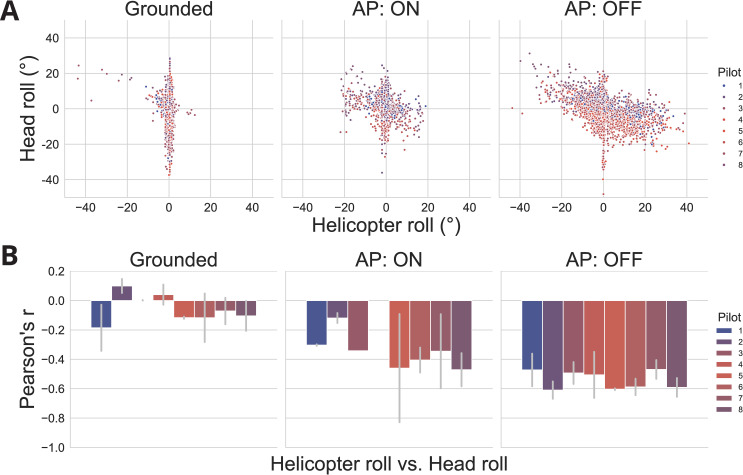
Anticorrelation between helicopter and head roll movements during flight, across different conditions of autopilot (AP). (**A**) scatter plot of helicopter and head roll movements. The figure shows only 1% of all available data, or roughly 17,000 pairs of values, sampled randomly amongst all pilots and scenarios. (**B**) Pearson correlation coefficient between helicopter and head roll movements.

What could account for such a simplification of the aircraft’s capabilities? Given the recent advances in the field of tool embodiment, we wondered whether this mechanism was a tactic implemented by the pilots, deliberately or otherwise, to finely grasp the motor-to-mechanical transformations [10] required to fly the helicopter, and in turn to be able to predict their sensory consequences. To investigate this issue, we compared the head movements of the pilots with the motion of the helicopter in the roll plane in different flight phases (cf., Material and methods). Indeed, we noticed a natural tendency for the pilots to try to maintain their visual field leveled with the external environment, which requires adequate counter-rotation of the head during roll motions of the aircraft. Fig 3 presents the correlation between helicopter motion and head movements.

The 2-dimensional graph of the roll motion of the helicopter versus roll movements of the head shows a linear trend when the helicopter is off the ground (Fig 3, A). When grounded, helicopter movement is theoretically prohibited. In practice though, artefactual samples appear on the figure due to temporal imprecision in phase definition: the *grounded* flight phases may briefly overlap with the following phase, which contains movement. This effect is nevertheless of negligible significance and does not undermine the overall value of this control condition.

The strength of the linear trends was quantified using the Pearson correlation coefficient (Fig 3, B). Compared to the control condition, both flying phases showed a significant anti-correlation between helicopter motion and head movement in the roll plane (p<0.01, Mann-Whitney U test). Statistical analysis reveals a stronger correlation when pilots are in full control of their aircraft (AP OFF condition) than when the automatic pilot is active (p<0.01, Mann-Whitney U test).

Our findings suggest that the pilots were able to assimilate the relationship that transforms a movement of the manipulandum in the roll plane into a motor command of the head that counteracts the aircraft’s inclination.

### Vestibular stimuli

How can such an intricate understanding of motor-to-mechanical transformations and their physiological consequences be achieved? For head movements, it is commonly accepted that both the active motion of the head and the biomechanical filtering properties of the body shape the structure of natural vestibular stimuli [26]. However, it is unclear how the characteristics of vestibular inputs are altered during a manual navigation task. In other words, piloting a helicopter induces additional vestibular information caused by the movements of the cabin. Correctly predicting the appropriate counter-rotation of the head during roll movements of the aircraft may therefore prove challenging given these non-ecological conditions.

**Fig 4:**
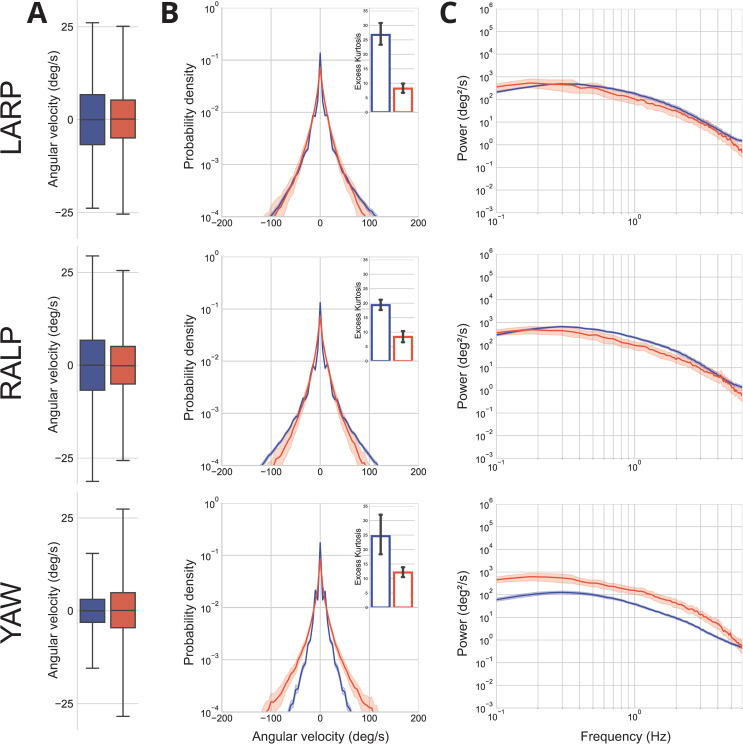
Comparison of the vestibular stimuli experienced by healthy subjects in natural, seated conditions and while piloting a helicopter. Head angular velocity was projected in the three planes of the semicircular canals: the left anterior, right posterior (LARP), right anterior, left posterior (RALP), and yaw (YAW) planes. (**A**) Boxplot of the population-averaged angular velocity signals for the helicopter pilots (blue) and control subjects (red). Boxes and whiskers correspond to 75% and 95%, respectively, of the data. Outliers are not represented. (**B**) Population-averaged probability density functions for the head velocity signals in the two activities with corresponding SD (shaded areas). Insets: population-averaged excess kurtosis values. The error bars represent the 95% confidence interval. (**C**) Population-averaged power spectra of the head angular velocity in the two tasks with corresponding 95% confidence interval (shaded areas).

The statistics of vestibular information during different ecological activities were investigated in a prior work [23]. In particular, the pilots of the present study were compared to a control condition of visual exploration in a seated posture. Fig 4 synthesizes the results.

We found no statistical difference between the velocity distribution (Fig 4, A) of the head movement in the two activities in all planes of the semicircular canals (p>0.05, Mann-Whitney U test, although we note a *p* -value p≈0.05 in the YAW plane). The probability density functions of the head angular velocity signals (Fig 4, B) differed significantly from the Gaussian distribution (p<0.01, Mann-Whitney U test), and the distribution in the manual navigation task was significantly more leptokurtic than in the control (p<0.01, kurtosis values, Mann-Whitney U test). The power spectral density (Fig 4, C) of the head angular velocity is qualitatively similar in the two activities, except for the YAW plane which shows a drop in the total power density. When compared quantitatively, statistical tests show that the total power in each plane is significantly different between the two tasks (p<0.01 in all planes, Mann-Whitney U test).

Taken together, our results indicate that although the frequency content of vestibular stimuli experienced by helicopter pilots in a simulator differs significantly from a natural sitting task, they share overall comparable properties. We posit that the integration of the machine’s laws of motion is facilitated by the restriction of movement to a range that only provides sensory feedback similar to those experienced in everyday situations.

## Discussion

The present study examined the characteristics of the interaction between human participants and a complex human-machine interface. Specifically, pilots performed flights in a highly realistic simulator. By analyzing the primary directions of motion of the aircraft, we found that pilots drastically constrained its theoretical range of motion. This effect was at least partly a product of the stereotypical movements of the control manipulandum, which were predominantly oriented along the cardinal directions. In this restricted context, we found a propensity to maintain the visual field level with the external environment by counter-rotation of the head during roll movements of the helicopter. Furthermore, we found that the statistics of the vestibular stimuli experienced during the flights were similar to those experienced in everyday life. Overall, our findings suggest that effective control of complex human-machine interfaces stems from a simplification of the machine’s capabilities: they operate within an anthropomorphic range to ensure sufficient sensorimotor integration and thus proper control.

### The law of interaction between a complex human-machine interface and its operator

Adaptive behavior relies on intricate neural processing within multiple interconnected networks encompassing both motor and sensory systems. This processing involves the use of efferent copies and internal models to predict and interpret sensory feedback resulting from self-generated movements and external stimuli. Efference copies forecast the sensory implications of a particular action and, consequently, allow the differentiation between self-generated (reafferent) sensory input and extrinsic sensory inflow [27]. They play a pivotal role in driving internal models, which generate prediction about the sensory consequences of planned actions [28]. By comparing the predicted and actual sensory feedback, the brain can monitor and adjust ongoing movements, facilitating smooth and coordinated behavior. This interplay between efferent copies, internal models, and sensory feedback is crucial for motor learning, sensorimotor adaptation, and the ability to interact with dynamic environments. These mechanisms also apply during the manipulations of tools. Given the growth in the complexity of machines over the past century [17,18], the question of the conceptual limits of such models has become increasingly salient. In other words, is it biologically plausible for the central nervous system to develop accurate hybrid internal models fusing the characteristics of complex machines, such as helicopters, and their operators? Empirical evidence indicates that pilots have the capacity to achieve mastery in the control of their aircraft, despite the inherent complexity of the aircraft itself. Nevertheless, our results suggest the use of a simplification strategy. Participants acted on their machine in either the roll or sagittal plane, but very rarely in both planes simultaneously. On a larger scale, helicopter motion was also strictly constrained compared to its theoretical possibilities.

### Anthropomorphism as a simplification mechanism

How is this simplification achieved? Our findings suggest that its goal is to emulate the characteristics of human motor control in typical conditions. The helicopter’s movements, in terms of orientation and magnitude, are strikingly similar to the properties of natural human head movements during locomotion. For instance, Einhäuser and colleagues [29] found that gaze, eye and head moved preferentially along the cardinal axes. In a virtual reality setting, Hu et al. [30] found that most head movements during a visual exploration task also occurred along the cardinal directions. Our results present a similar pattern: helicopter motion is predominantly oriented in the cardinal directions with limited vertical movements, large outgrowths in the horizontal plane and only few oblique tilts. The excursions observed in the helicopter’s flight envelopes further match the statistics of human head orientation during natural unconstrained activities. In particular, Sinnott and colleagues [31] recently reported larger distributions of head roll than head pitch (as evidenced by much greater excess kurtosis values). The head pitch distribution was asymmetric with an over-representation of downward head tilt, consistent with ground orientation behavior. Analogous findings can be observed in Fig 1, although we wish to point out that the symmetry observed in the roll plane may be context-dependent, i.e., situational factors may favor one direction of movement over the other in different flight scenarios. It should also be noted that the much smaller range of motion in the vertical plane may be partly attributed to the customary piloting procedures: air traffic regulations require that certain cruising altitudes are maintained, hence pure altitude changes without simultaneous movement in the horizontal plane are generally only observed during take-offs and landings and are therefore quite rare.

Studies using multidirectional perturbations have shown that postural control in humans [32–34] and in animal models [35] is distinct in the roll and pitch planes. This is largely due to biomechanical constraints. Motor control in the roll plane requires more complex asymmetrical coordination of muscle responses and probably places greater demands on the output processing requirements of the CNS than those required for symmetrical muscle patterns in the sagittal plane. In the present study, pilots mostly controlled the aircraft using successive motion in the yaw, pitch or roll spaces, which resulted in very few oblique trajectories that were mostly observed for small movements. This phenomenon is evocative of the segregation of motor control in human. In addition, while the manual manipulation of the joystick controls both the movements in the pitch and roll planes, control of the yaw plane is implemented separately by two pedals. That design choice is amusing as it echoes vertebrate evolution, during which mobility in the yaw plane appeared later than in the pitch and roll planes, and therefore uses distinct neural structures.

### *Enmachinement: sensorimotor* integration through simplification

Human tool-use proficiency is thought to be the result of our ability to incorporate tools into a multisensory body representation [36]. The question of whether tool use alters the body schema remains a subject of contention within the field [37]. Regardless, the concept of tool-embodiment entails the development of hybrid internal models that include external objects into the body to model the dynamics of the body supplemented by this object. In the remainder of the present discussion, we propose to use the term enmachinement to distinguish the case where the object to control is a complex machine instead of a simple tool operated by hand. Prior research on the impact of tool complexity on the cognitive aspects of tool use has provided contradictory findings. While active tool use was found to modulate sensorimotor experience during simple repetitive reaching [38], increasing the difficulty of the task, for instance retrieving and sorting beanbags [39], was shown to reduce the effects of embodiment [40]. Our findings suggest some level of prediction of the sensory consequences of the actions on the commands. Observation of the pilots’ head movements in the roll plane reveals a natural tendency for the pilots to try to keep their visual field leveled with the external environment, requiring adequate counter-rotation of the head during roll movements of the aircraft. Overall, helicopter flight envelope limitations and head stabilization in the roll plane would aim to keep angular head accelerations within the operating range of the vestibulo-ocular and optokinetic reflexes (VOR) to preserve pilots’ visual acuity. This requires the pilot to predict the motor-to-mechanical transformations [10] and how the latter will affect their sensory organs. A detailed analysis of the frequency content of vestibular information experienced by the pilots during their flight shows strong similarities with the frequency spectra measured in seated participants during a simple gaze orientation task. In other words, the pilots restricted the helicopter’s movements so that visual, vestibular and proprioceptive afferents mimicked those on the ground, further corroborating enmachinement. Recent research in the field of mental workload estimation with machine learning models also point in this direction.

### Quantification of perceptual-motor style through enmachinement

Even stereotyped behavior is subject to variability due to noise, redundancy, adaptability, learning or plasticity. The idea that some of this variability depends on unique individual strategies has recently gained traction in the literature under the concept of perceptual-motor style (see Vidal and Lacquaniti [41] for a review). While this study has identified a number of general trends in the control of complex human-machine interfaces, our findings also uncovered considerable inter-pilot variability in their use of helicopter controls. In particular, metrics derived from the two-dimensional motion of the manipulandum (in the pitch and roll planes) may be important markers for determining pilots’ perceptual-motor style: some, such as the force applied on the manipulandum, show low intra-pilot variability but high inter-pilot variability in our dataset (results not shown here). This methodology could be employed systemically to pioneer automatic pilot identification or the early detection of physiological and/or cognitive impairments during flight. For instance, Bonfati et al. [42] demonstrated a strong correlation between terrestrial vehicle data and physiological data from the driver, which suggests a promising avenue for future research, namely restricting the analysis to machine-generated data to characterize the behavior of its operator. In that regard, a recent study by Keriven Serpollet et al. (submitted), trained a machine learning model with the physiological and operational parameters recorded during flight scenarios to predict the pilots’ mental load and showed that operational information outperformed physiological signals in terms of their predictive power. This supports the hypothesis that pilots “integrate” the helicopter, in the sense that its operational characteristics accurately reflect their own mental workload.

### Limitations

This study is subject to several limitations. As is the case with any work conducted in a natural setting, measurement of human behavior is prone to noise, which affects the quality of the recorded signals. Due to limitations in pilot availability, the number of participants included in the experiment is relatively small and the study population is very homogeneous (only men, all professional pilots). Finally, it should be noted that piloting a helicopter simulator is likely to differ from piloting in the real world. Specifically, prior research has found that while some operational characteristics remained nearly identical in simulated vs. real conditions, other cognitive aspects, such as subjective workload ratings, may vary [43].

## Conclusion

The operation of a complex human-machine interface, such as that required to pilot an aircraft, places a significant mental burden on the operator due to the numerous degrees of freedom and inherent complexity of the machine. Indeed, the degrees of freedom may exceed the operator’s ability to generate the hybrid internal model required to control the machine, making the process of acquiring proficiency with the tool fragile, if not impossible. Our findings suggest a simple solution to this problem: the operators limit the machine’s capabilities, i.e., they simplify its theoretical operational envelope to render its control feasible. To achieve this goal, several results of this study point to a control scheme whose rules are largely inspired by the laws that govern human movement, that is, by anthropomorphizing the machine. Such a simplification within an anthropomorphic range facilitates its sensorimotor integration.

An interesting avenue of research is therefore the development of human-machine interfaces adapted to the limits of human cognitive capacity. Moreover, it is fundamental to study the consequences of using a complex tool outside of a simplistic operating regime. This includes investigating the point at which the development of an internal model becomes infeasible and the subsequent repercussions.

## Supporting information

S1 Fig Simulator and scenario information. (PDF)

S2 Fig Visual representation of pilot information. (PDF)

S3 Fig Trajectory segmentation method. (PDF)

S4 Fig Processing of the manipulandum data. (PDF)
