# Supplementary material for: Beware of physiology: Anthropomorphism as a simplification mechanism for mastering complex human-machine interfaces
Source: PLoS One. 2025 Apr 15;20(4):e0321580. doi: 10.1371/journal.pone.0321580 (PMC11999125; doi:10.1371/journal.pone.0321580)
Supplement: S1 Fig — (PDF) [file pone.0321580.s001.pdf]

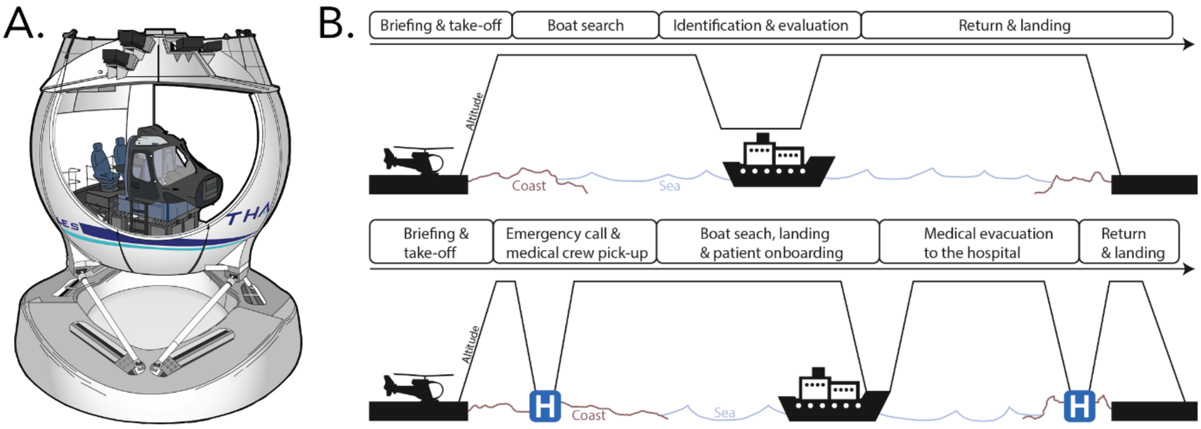

S1 Fig. Simulator and scenario information*. A: Full-flight simulator. The EC135 helicopter cabin is located inside a display dome, controlled by a 6-degree-of-freedom platform. B: Sequence of tasks in the first (top) and second (bottom) scenarios. Pilots were asked to conduct two realistic scenarios in the simulator. In the first scenario (approximately 1 hour), pilots were tasked to research and identify a boat that was spilling oil at sea. After the briefing, they flew off the coast, searched for the boat, identified the boat registration, and returned. In the second scenario (approximately 1 hour and 30 minutes), pilots embarked on a rescue mission at sea. After take-off, they received an emergency call and were commanded to intervene. To do so, they had to adapt their flight plan, pick up a medical crew at the hospital, fly towards the boat, and land. With the wounded individual now on board, pilots made their way back to the hospital. However, before reaching their destination, they were asked to land in a low-visibility area for an emergency landing because the condition of the patient was deteriorating. After this eventual stop, pilots dropped off the medical team at the hospital and returned to the airport.*
