# Supplementary material for: Beware of physiology: Anthropomorphism as a simplification mechanism for mastering complex human-machine interfaces
Source: PLoS One. 2025 Apr 15;20(4):e0321580. doi: 10.1371/journal.pone.0321580 (PMC11999125; doi:10.1371/journal.pone.0321580)
Supplement: S2 Fig — (PDF) [file pone.0321580.s002.pdf]

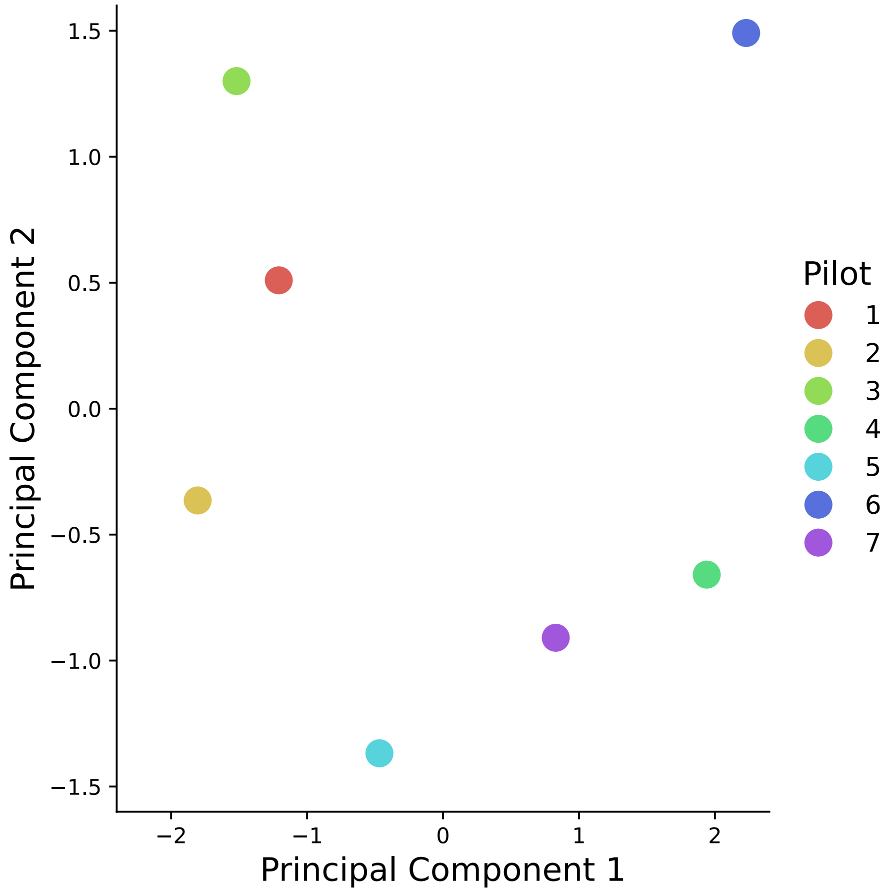

S2 Fig. Visual representation of pilot information. *A principal component analysis was conducted with the age, hours of total real flight experience, hours of real flight experience in the last twelve months, and hours of simulator experience of pilots 1 through 7 for visualization purposes. Data from pilot 8 was not considered: it is an outlier with extensive experience in both real and simulated helicopters. The first two principal components explain 86% of the variance. The real flight experience contribute the most to the first component, while the age and simulator experience contribute most to the second component. The scatter plot of these two components indicates a good coverage of the space of pilot profiles.*
