# Supplementary material for: Beware of physiology: Anthropomorphism as a simplification mechanism for mastering complex human-machine interfaces
Source: PLoS One. 2025 Apr 15;20(4):e0321580. doi: 10.1371/journal.pone.0321580 (PMC11999125; doi:10.1371/journal.pone.0321580)
Supplement: S3 Fig — (PDF) [file pone.0321580.s003.pdf]

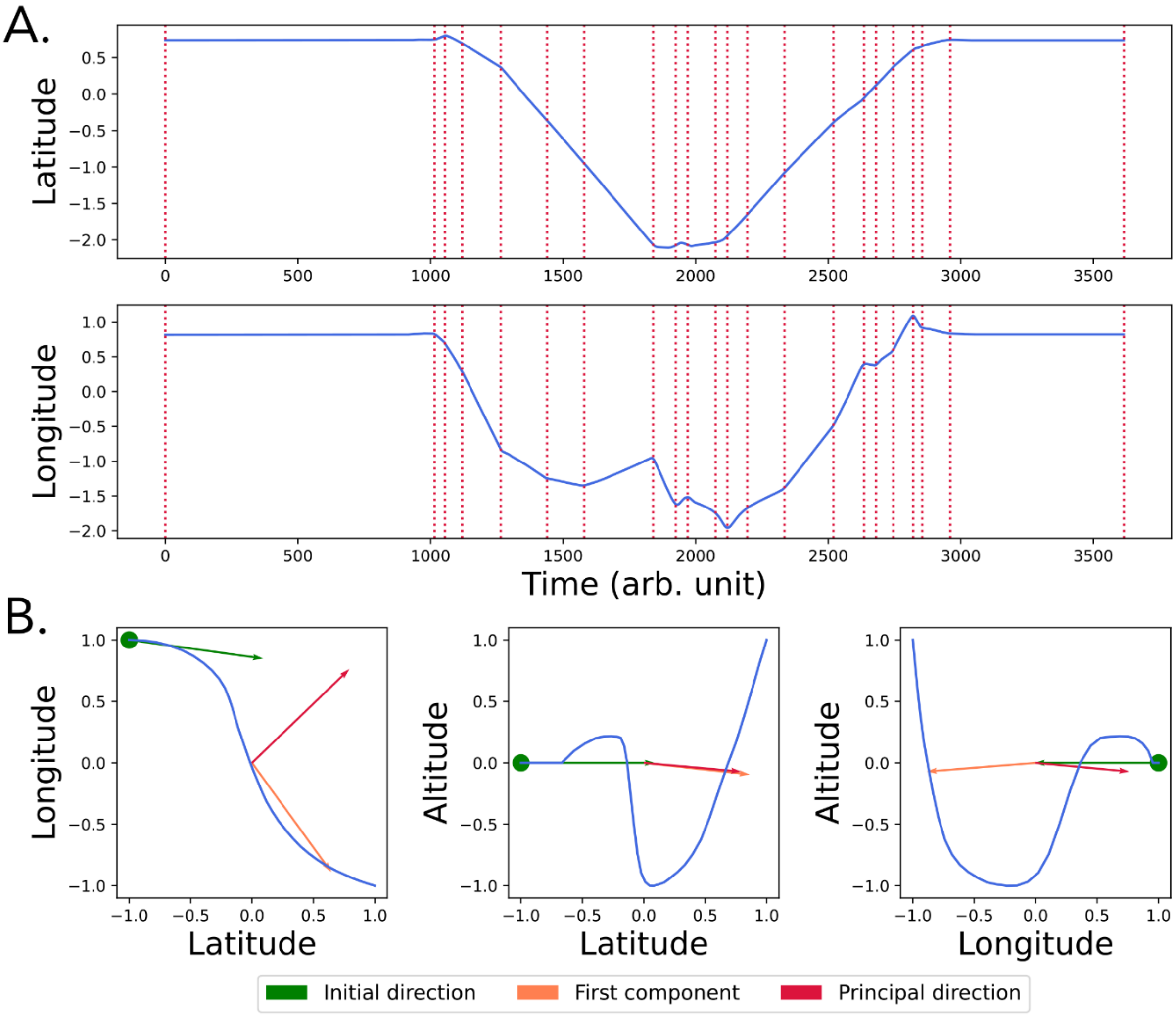

S3 Fig. Trajectory segmentation method. *A: Results from the change point detection algorithm (Pelt, $model = clinear$, $n_{min} = 10$) for the second pilot in the first scenario. Change points were detected in the 2-dimensional array consisting of the latitude and longitude data and are represented by the red, dotted vertical lines. B: Identification of the principal direction of movement in a flight segment. The blue curve represents the trajectory of the aircraft. The green vector corresponds to the initial direction of the helicopter in this segment. The orange vector shows the first component of the principal component analysis. The red vector illustrates the resulting principal direction, obtained from the difference between the first component and the initial direction, followed by an arbitrary reorientation so that the vector is expressed in the orthonormal reference framed generated by the latitude, longitude, and altitude axes.*
