# Supplementary material for: Beware of physiology: Anthropomorphism as a simplification mechanism for mastering complex human-machine interfaces
Source: PLoS One. 2025 Apr 15;20(4):e0321580. doi: 10.1371/journal.pone.0321580 (PMC11999125; doi:10.1371/journal.pone.0321580)
Supplement: S4 Fig — (PDF) [file pone.0321580.s004.pdf]

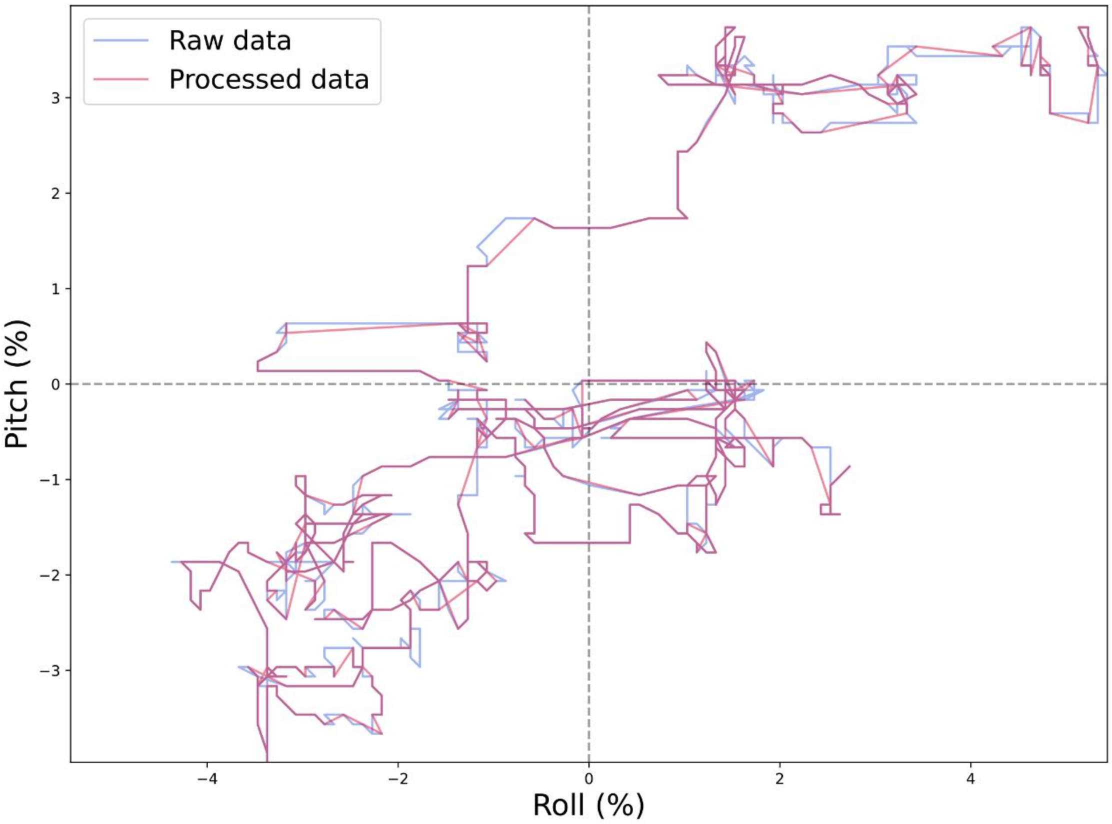

S4 Fig. Processing of the manipulandum data. *Segmentation of the cyclic stick's motion. Successive samples were concatenated together into segments according to an angular deviation threshold set to* $\tau_{angle} = 10$ *degrees.*
